# Neutrophil-to-lymphocyte ratio as a predictive biomarker for moderate-severe ARDS in severe COVID-19 patients

**DOI:** 10.1186/s13054-020-03007-0

**Published:** 2020-06-05

**Authors:** Aijia Ma, Jiangli Cheng, Jing Yang, Meiling Dong, Xuelian Liao, Yan Kang

**Affiliations:** Department of Critical Care Medicine, West China Hospital of Sichuan University, No. 37, Guoxue Alley, Chengdu, 610041 Sichuan Province China

## Abstract

ChiCTR, ChiCTR2000029758. Registered 12 February 2020 - Retrospectively registered

Dear editors:

The COVID-19 pandemic has spread rapidly around the world and overwhelmed the supply of intensive care beds and ventilators; judicious ICU resource allocation is still one of the major challenges for clinicians and management [1]. The higher incidence of ARDS is the main reason for the burden of ventilator equipment. Early prediction of the occurrence and aggravation of ARDS in the ICU helps clinicians prepare for respiratory support equipment given the absence of effective treatment strategies. Moreover, early selected patients with severe ARDS who do not benefit from conventional treatment might be successfully supported with V-V ECMO [2], which is a relatively scarce critical care resource. Therefore, early prediction of moderate-severe ARDS can help clinicians better allocate scarce ICU resources for COVID-19 crisis.

Neutrophil-to-lymphocyte ratio (NLR) is a simple biomarker of inflammation that can be measured during routine hematology. Previous studies have exhibited that higher NLR was associated with clinical deterioration and mortality for COVID-19 patients [3]. However, it remains unclear to what extent the significance of NLR would predict the occurrence of ARDS and ICU ventilator requirements for the COVID-19 crisis.

Patients diagnosed with severe COVID-19 from 21 hospitals in Sichuan Province between January 16 and March 15 were included in the analysis (ChiCTR2000029758). The maximum value of NLR, PLR, PCT, and CRP during the first 3 days after being diagnosed as severe COVID-19 was included in the analysis. Severe COVID-19 and ARDS were defined according to previous study [4] and Berlin definition [5], respectively. Multivariate logistic regression analysis and the area under the receiver operating characteristic (ROC) curve were used to analyze the ability of NLR in predicting ARDS.

Of totally 81 patients defined as severe COVID-19, 44 were diagnosed as ARDS. The baseline characteristics of the non-ARDS group and ARDS group are listed in Table 1. The area under the ROC curve for ARDS was 0.71, 0.591, 0.494, and 0.625 for NLR, PLR, PCT, and CRP, respectively. We used the median as the cutoff value to divide the patients into two groups. The high NLR group (NLR > 9.8) showed a higher incidence of ARDS (*P* = 0.005) and higher rate of noninvasive (*P* = 0.002) and invasive (*P* = 0.048) mechanical ventilation. Further, we defined moderate-severe ARDS as ARDS patients with oxygenation index less than 150. The area under the ROC curve for moderate-severe ARDS was 0.749, 0.660, 0.531, and 0.635 for NLR, PLR, PCT, and CRP, respectively (Fig. 1); the cutoff value of NLR for moderate-severe ARDS is 11.

**Fig. 1:**
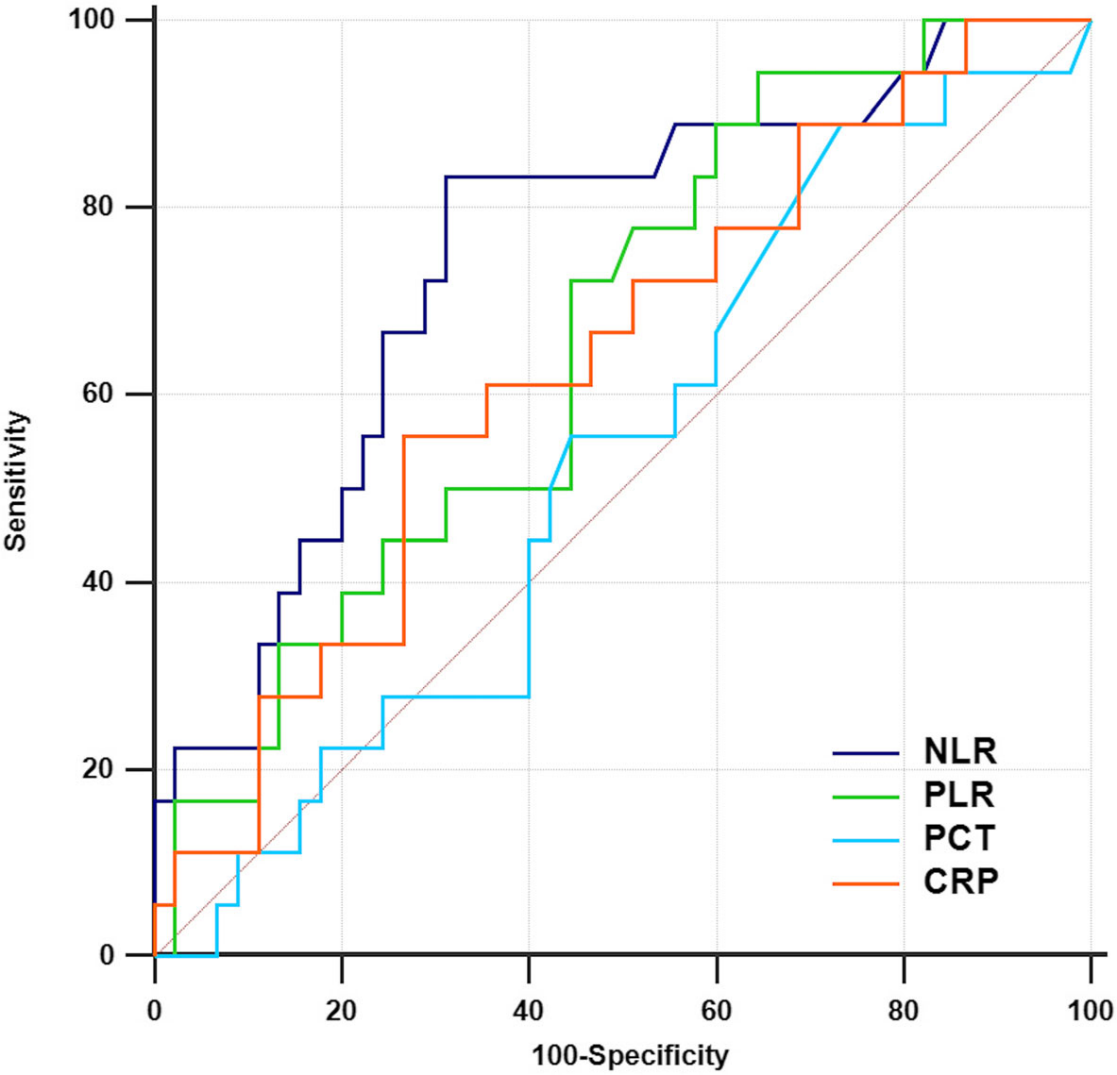
Moderate-severe ARDS prediction biomarkers in severe COVID-19 patients: NLR (0.749, 95% CI 0.624–0.850), PLR (0.660, 95% CI 0.530–0.775), PCT (0.531, 95% CI 0.401–0.658), and CRP (0.635, 95% CI 0.504–0.752)

**Table 1:** Baseline characteristics and clinical outcomes stratified by median NLR value

**Baseline characteristics**	**Non-ARDS*N*** **= 37**	**ARDS*N*** **= 44**	***P*values**
Age	49 (36.5–62.5)	53.5(43–70.5)	0.110
Gender/case (%)			0.891
Male	23 (62.3%)	28 (63.6%)	
Female	14 (37.8%)	16 (36.4%)	
BMI (kg/m^2^)	23.05 (22.00–27.25)	24.78(21.29–27.41)	0.816
Smoking/case (%)	1 (2.7%)	2 (4.5%)	1.000
Comorbidities/case (%)			
Diabetes	3 (8.1%)	15 (34.15)	**0.007**
Hypertension	7 (18.9%)	8 (18.2%)	0.932
Chronic pulmonary disease	2 (5.4%)	9 (20.5%)	**0.049**
Cardiovascular disease	2 (5.4%)	2 (4.5%)	1.000
Cerebrovascular disease	0 (0%)	3 (6.8%)	0.246
Renal disease	1 (2.7%)	2 (4.5%)	1.000
Liver disease	2 (5.4%)	2 (4.5%)	1.000
Vital signs			
MAP/mmHg	94.67 (89.17–100.50)	97.83(91.75,108.84)	0.162
Heart rate (beats/min)	88 (77.5–99)	92.5 (85.25–104)	0.175
Respiratory rate (breaths/min)	20 (20–22.5)	21 (20–23)	0.107
Pulse oxygen saturation/%	96 (93.75–97.25)	95 (90.25–97)	0.486
Laboratory findings			
WBC/10^9^/L	5.43 (4.05–6.59)	6.47 (3.94–9.62)	0.122
Hemoglobin/g/L	141 (127–153.5)	132 (117.25–146.5)	0.107
Total bilirubin (μmol/L)	9 (5.93–15.6)	9.3 (6.65–14.3)	0.927
AST (IU/L)	30.5 (19–39.75)	29.15 (15.75–57.68)	0.764
ALT (IU/L)	30 (25–39.8)	35 (25.75–51.6)	0.221
Creatinine (μmol/L)	71.75 (54.35–79.75)	69.2 (54.63–80.53)	0.980
PT/s	12.7 (12.5–13.98)	13.1 (12.6–13.8)	0.787
APTT/s	32.75 (29.1–40.13)	31.3 (28.8–35.5)	0.246
NLR/%	6.4 (3.75–13.1)	13.55 (6.05–24.13)	**0.002**
**Clinical outcomes**	**Low NLR*N*** **= 41**	**High NLR*N*** **= 40**	***P*value**
Respiratory support			
High-flow nasal cannula	15 (36.6%)	16 (40%)	0.752
Noninvasive ventilation	5 (12.2%)	17 (42.5%)	**0.002**
Invasive ventilation	2 (4.9%)	8 (20%)	**0.048**
ARDS			
Mild-moderate ARDS	11 (26.8%)	11 (27.5%)	0.946
Moderate-severe ARDS	5 (12.2%)	11 (42.5%)	**0.002**

Data are presented as interquartile range or number (percentage)

*BMI* body mass index, *MAP* mean arterial pressure, *WBC* white blood cell, *AST* aspartate aminotransferase, *ALT* alanine aminotransferase, *PT* prothrombin time, *APTT* activated partial thromboplastin time, *NLR* neutrophil-to-lymphocyte ratio, *ARDS* acute respiratory distress syndrome

Our data revealed that NLR could be a valuable biomarker to recognize severe COVID-19 patients with moderate-severe ARDS, which facilitated clinicians to give effective respiratory supporting strategies and quickly find out moderate-severe ARDS patients who are at high indication for V-V ECMO.

Because of the mismatch of the oxygenation and lung function [6], a comprehensive consideration of immune indicators would improve early prediction for COVID-19 patients with “atypical” ARDS [6]. NLR is an extremely common laboratory test wherein the initial NLR value can be used to identify high-risk patients with moderate-severe ARDS, with the optimal threshold value of 11. This biomarker may be helpful in assessing the allocation of respiratory equipment in ICU patients and early assessment of ECMO. However, further clinical studies are needed to evaluate the benefits of NLR in ARDS.

## Data Availability

The datasets used for the analysis in the current study are available from the corresponding author on reasonable request.

## Abbreviations

COVID-19: Coronavirus disease 2019
ARDS: Acute respiratory distress syndrome
ICU: Intensive care unit
WHO: World Health Organization
ECMO: Extracorporeal membrane oxygenation
V-V ECMO: Veno-venous extracorporeal membrane oxygenation
NLR: Neutrophil-to-lymphocyte ratio
PLR: Platelet-to-lymphocyte ratio
PCT: Procalcitonin
CRP: C-reactive protein
ROC: Receiver operating characteristic

## References

[1] Liew MF, Siow WT, MacLaren G, See KC (2020). Preparing for COVID-19: early experience from an intensive care unit in Singapore. Crit care (London).

[2] Ramanathan K, Antognini D, Combes A, Paden M (2020). Planning and provision of ECMO services for severe ARDS during the COVID-19 pandemic and other outbreaks of emerging infectious diseases. Lancet Respir Med.

[3] Liu Y, Du X, Chen J, et al. Neutrophil-to-lymphocyte ratio as an independent risk factor for mortality in hospitalized patients with COVID-19. J Infect. 2020;S0163-4453(20)30208-5. 10.1016/j.jinf.2020.04.002. 10.1016/j.jinf.2020.04.002 PMC7195072 32283162

[4] Liao X, Chen H, Wang B, Li Z, et al. Critical care for patients with severe COVID-2019 in Sichuan Province, China——a provincial cohort study.medRxiv 2020.03.22.20041277; 10.1101/2020.03.22.20041277.

[5] Force ADT, Ranieri VM, Rubenfeld GD, Thompson BT (2012). Acute respiratory distress syndrome: the Berlin definition. Jama.

[6] Gattinoni L, Coppola S, Cressoni M, et al. COVID-19 Does Not Lead to a “typical” acute respiratory distress syndrome. Am J Respir Crit Care Med. 2020;201(10):1299–300. 10.1164/rccm.202003-0817LE. 10.1164/rccm.202003-0817LE PMC7233352 32228035

